# Prevalence and factors associated with uncontrolled type 2 diabetes among patients in Kirehe District, Rwanda: a cross-sectional study

**DOI:** 10.11604/pamj.2024.48.128.43864

**Published:** 2024-07-24

**Authors:** Symaque Dusabeyezu, Jean Nepomuscene Renzaho, Nasiru Sani

**Affiliations:** 1 Department of Public Health, Mount Kenya University Rwanda, Kicukiro District, Kigali City, Rwanda,; 2 Department of Non-Communicable Diseases, Partners in Health, Inshuti Mu Buzima, Gasabo District, Kigali City, Rwanda,; 3 Department of Research and Training, Partners in Health, Inshuti Mu Buzima, Gasabo District, Kigali City, Rwanda,; 4 University of Rwanda, Kicukiro District, Kigali City, Rwanda

**Keywords:** Prevalence, factors, HbA1c, uncontrolled type 2 diabetes mellitus

## Abstract

**Introduction:**

the increase in type 2 diabetes mellitus (T2DM) is a major public health issue on a global scale. A continuous rise in blood sugar levels, even if there are no symptoms of diabetes, leads to tissue degeneration and, in certain cases, fatal diseases.

**Methods:**

in this cross-sectional study, the researcher examined the prevalence of uncontrolled type 2 diabetes mellitus among patients in Kirehe District Health Facilities from October 2023 to February 2024. The target population was 333 patients and the sample size was 201 patients who visited the non-communicable disease clinics throughout the time of data collection, those with T2DM diagnosis for at least a year, and non-pregnant women.

**Results:**

the mean age was 57.23 years with an 11.06 standard deviation. Females represented 58.7% (n=118) while males were 41.3% (n=83). The prevalence of uncontrolled type 2 diabetes mellitus was 72.6% (n=146). Patients who had ever been lost to follow-up were more likely to have uncontrolled T2DM (AOR=3.24, 95% CI: 1.06-5.90, p=0.036) compared to those who regularly visited the health facility for care and follow-up. Patients who had comorbidities to diabetes were more likely to have uncontrolled T2DM (AOR=2.48, 95% CI: 1.31-4.68, p=0.005) compared to those who did not have comorbidities.

**Conclusion:**

the prevalence of uncontrolled T2DM is high; healthcare providers have a responsibility to perform home visits to reduce the lost to follow-up rate and to conduct regular screening sessions for diabetes comorbidities.

## Introduction

Type 2 diabetes affects 537 million people worldwide, with a prevalence of 10.5%. About 6% of the deaths that occurred in Social Security Administration (SSA) in 2017 were attributed to diabetes, which is estimated to go undetected in more than 69% of diagnosed adults [1]. More than one million deaths per year are solely attributable to diabetes, making it the 19^th^ most common cause of death on a global scale [2]. The prevalence of type 2 diabetes is rising globally, and in developed nations like Western Europe, the disease is emerging more quickly [3]. Without adequate action being made to address the issue, it is predicted that by 2030, the prevalence of diabetes will be 11.3%, or 643 million people, and if trends continue, by 2045, the figure will rise to a startling 783 million (12.2%), echoing a global trend [4]. Compared to high-income nations, the burden of type 2 diabetes has increased more quickly in low- and middle-income countries in terms of both prevalence and the number of persons affected [5]. Initiatives for clinical preventive and public health are crucial [6].

According to James M *et al*. [7], the prevalence of diabetes was found between 3.1 and 4.3%. A patient whose average glycemia over three consecutive visits exceeds 140 mg/dL or 7.8 mmol/L, or whose H (HbA1c) exceeds 7%, is considered to have poor glucose control [8]. Research at 49 private clinics that treat diabetic patients in Malaysia discovered that 20% of patients had HbA1c levels below 7% and FBG levels below 7.8 mml/L levels below 25%. According to a study conducted in Malaysia, 65 percent of type 2 diabetes patients had uncontrolled diabetes [9]. Other studies conducted in various countries, including Ghana, Nigeria, Bangladesh, Sudan, Zambia, and Thailand, have revealed an increase in the prevalence of type 2 diabetes without treatment. The rates were 86.4%, 83.3%, 82.0%, 80.0%, 61.3%, and 79.64%, respectively [10]. In Kenya, the prevalence of uncontrolled T2DM was 93% according to the study done in 2018 [11]. The studies conducted in many countries such as Ghana, Nigeria, Bangladesh, Sudan, Zambia, and Thailand showed that the prevalence of uncontrolled diabetes is increased by 86.4%, 83.3%, 82.0%, 80.0%, 61.3%, and 79.64, respectively, and the glycemic control was 20% in other countries, such as Mali, with an HbA1c of 7% and fasting blood glucose of 7.8 mmol/L at 25% [10]. In Kenya, the prevalence was high (93%) in a study conducted in 2018 [11]. This study aimed to determine the prevalence of uncontrolled T2DM and identify its associated factors among patients in Kirehe District, Rwanda.

## Methods

**Study design and setting:** this cross-sectional study employed a quantitative methodology to determine the prevalence and factors associated with uncontrolled type 2 diabetes mellitus among patients in Kirehe District Health Facilities during a period from October 2023 to February 2024. The study was conducted in 18 health facilities of Kirehe District: Kirehe District Hospital and 17 Health Centers; Bukora, Gahara, Gashongora, Kabuye, Kigarama, Kigina, Kirehe, Mahama, Mulindi, Musaza, Mushikiri, Nasho, Ntaruka, Nyabitare, Nyarubuye, Rusumo and Rwantonde. Kirehe District is one of the seven Districts that compose the Eastern Province of Rwanda. Rwanda is composed of 30 Districts that are grouped into four Provinces (East, North, West, South, and Kigali) and Kigali City. Rwanda is located in East Africa with four neighboring countries: Uganda in the North, Burundi in the South, DR Congo in the West, and Tanzania in the East.

**Target population and sample size:** this study included all patients with T2DM aged 18 and up who were being treated in the Kirehe District Health Facilities in the Eastern Province of Rwanda. All health facilities were following 333 patients with T2DM at the end of February 2024 according to Kirehe District Health Management Information System. The inclusion criteria were: all patients who have had a type 2 diabetes diagnosis for at least a year who visited NCD clinics during the study period while patients who refused to participate in the study, those who did not visit the non-communicable disease clinics throughout the time of data collection, those with T2DM diagnosis for less than a year and expectant women were excluded from the study. The sample size was 201 obtained by using Yamane Taro´s formula. In this study, the sample size was calculated using the Yamane Taro´s formula with a 95% confidence interval and a 5% margin of error; n= sample size; N= target population; e=margin of error.


$$n=\frac{N}{1+\left(N\left(e*e\right)\right)}$$

$$n=\frac{333}{1+\left(333\times \left(0.05*0.05\right)\right)}$$

The sample size was 182 participants plus 10% of this, the sample size was 201 patients. The sample size for each health facility was proportionally calculated. To select the study participants, stratified simple random sampling was applied.

**Data collection instrument and procedures:** the information was gathered via a structured questionnaire. The questionnaire was written in English and then translated into Kinyarwanda, the respondents' native language. The questionnaire was divided into three parts: The first one consisted of the socio-demographic characteristics of the participants, the second was dedicated to clinical characteristics of the participants and the last was the value of the HbA1c test to determine if the diabetes was controlled or not among study participants. At the time of enrollment, trained data collectors gathered demographic and socioeconomic data by administering structured questionnaires.

**Definitions:** the flowing are key variables for this study comprised three types of variables: independent, dependent and intervening variables. Independent variables were grouped into two categories: patient socio-demographics included age, sex, marital status, level of education, occupation, religion, health insurance, distance from home to health facility and level of income, and patient clinical characteristics that included type of medications, treatment length, defaulter status, patient comorbidities, physical exercise, compliance to medication. The dependent variable was uncontrolled T2DM (HbA1c ≥7% or fasting blood sugar >200mg/dl) while the intervening variables were health education and Ministry of Health Policy to prevent uncontrolled T2DM.

**Data analysis:** following data collection, statistical package for the social sciences (SPSS) 21 software was used to enter and evaluate information from health facility records and questionnaire responses from respondents. Descriptive statistics were employed by frequency and percentage to ascertain the prevalence of uncontrolled T2DM. In order to determine the factors influencing patients' uncontrolled T2DM in Kirehe District Health Facilities, odds ratios with 95% confidence intervals were utilized. In order to determine the factors associated with uncontrolled T2DM, significant variables found by binary analysis were filtered to multivariable logistic regression, taking into account the adjusted odds ratio (AOR) and level of significance. The study's results were deemed significant when the p-value was less than 0.05. The study's findings were presented as a Word document with tables and text.

**Ethical considerations:** before conducting the study, the researchers sought Ethical Clearance from Mount Kenya University, Rwanda, the Institutional Review Board [REF: MKU/04/PGS&R/1161/2024] and approval from Kirehe District Hospital Leadership [REF NO: 080/HKIR/2024]. Prior to the interview, each study participant signed a consent form. The patients´ data were anonymous. Before the interview, the patients were informed about their voluntary participation in the study and ensured that there was no risk of refusing or withdrawing at any time during the interview. Also, they were informed that participation is just free. To access the secondary data, the hospital and health centers provided permission to data collectors to reach easily the participants and to conduct interviews.

## Results

**Socio-demographic characteristics of study participants:** study results revealed that most of the participants (44.3%) were aged between 41-51 years. The maximum and minimum ages were 90 and 27 years old, respectively, with 57 years old as the mean. Most of the participants were female (58.7%). A lot of them were married (70.6%). Most of them completed the primary education (62.2%). Most of the participants are live by cultivating (71.5%). A large number of participants adhere to Protestantism (46.8%). Only 1.5% do not have a health insurance. More participants took between one and two hours to reach the health facility to see care and according to the level of income, most of them were in the third category income level with 50.2% (Table 1).

**Table 1:** socio-demographic characteristics of study participants

Variables	Frequency (n=345)	Percentage (%)
**Age (Years)**		
15-40	13	6.4
41-56	89	44.3
57-72	87	43.3
73+	12	6.0
**Gender**		
Male	83	41.3
Female	118	58.7
**Marital status**		
Married	142	70.6
Single	5	2.5
Separated	7	3.5
Divorced	27	13.4
Widowed	20	10.0
**Level of education**		
Uneducated	44	21.9
Primary	125	62.2
Secondary	30	14.9
Above secondary	2	1.0
**Occupation**		
No occupation	17	8.5
Farmer	144	71.5
Merchant	9	4.5
Government employee	8	4.0
NGO employee	1	0.5
Casual laborer	7	3.5
Others	15	7.5
**Religion**		
Catholic	90	44.8
Protestant	94	46.8
Islam	6	3.0
No religion	11	5.6
**Health insurance**		
Yes	198	98.5
No	3	1.5
**Time to reach the health facility**		
<1 hour	198	32.3
1-2 hours	3	42.8
More than 2 hours	50	24.9
**Level of income in Rwanda**		
First category	24	12.0
Second category	76	37.8
Third category	101	50.2

**Participants´ clinical characteristics:** study findings showed that most of the patients (88.1%) were taking oral medications (tablets) for diabetes management. A lot of patients were on treatment for less than 5 years. According to defaulter status, 16.9% were lost to follow-up during the treatment period. Most of the patients (64.7%) said they had comorbidity to diabetes, most participants exercised physically and most (57.2%) were compliant with their diabetes medications (Table 2).

**Table 2:** participants’ clinical characteristics

Variables	Frequency (n=345)	Percentage (%)
**Types of medications**		
Insulin	15	7.5
Oral medications (tablets)	177	88.1
Insulin + oral medications	9	4.5
**Duration of the treatment**		
<5 years	90	44.8
5-10 years	75	37.3
More than 10 years	36	17.9
**Lost to follow up**		
Yes	34	16.9
No	167	83.1
**Any comorbidity?**		
Yes	130	64.7
No	71	35.3
**Physical exercise**		
Yes	90	44.8
No	111	55.2
**Compliance with medications**		
Yes	115	57.2
No	86	42.8

**Prevalence of uncontrolled T2DM among patients in Kirehe District Health Facilities:** of the total sample, 201 tested for HbA1c, 55 patients showed the results of HbA1c less than 7% while the rest, 146 patients had the level of HbA1c greater or equal to 7%. Therefore, the prevalence of uncontrolled type 2 diabetes mellitus among patients in Kirehe District of Rwanda was 72.6%.

**Multivariable analysis of factors associated with uncontrolled type 2 diabetes mellitus in Kirehe District of Rwanda:** factors analyzed in this section were only those that were found to be associated with uncontrolled T2DM based on the results of binary analysis. These factors include occupation of the patient (p=0.005), time used to reach the health facility (p=0.006), being lost to follow up at the health facility (p=0.008), having a comorbidity (p=0.005) and being compliant with medications (p=0.016). The above five factors were transferred to the multivariable analysis and two factors were significantly associated with the outcome variable; loss to follow-up at health facility and having a comorbidity were significantly associated with uncontrolled T2DM among patients in Kirehe District Health Facilities. Patients who had ever been lost to follow-up were more likely to have uncontrolled T2DM (AOR=3.24, 95% CI: 1.06-5.90, p=0.036) compared to those who regularly visited the health facility for care and follow-up. Patients who had comorbidities to diabetes were more likely to have uncontrolled T2DM (AOR=2.48, 95% CI: 1.31-4.68, p=0.005) compared to those who did not have comorbidities (Table 3).

**Table 3:** multivariable analysis of factors associated with uncontrolled T2DM in Kirehe District, Rwanda, 2024

Variables	Uncontrolled T2DM			
	Crude Odds ratio [95% CI]	p-value	Adjusted Odds ratio [95% CI]	p-value
**Occupation**				
No occupation	4.25 1.25-29.17)	0.097	3.61 (0.54-23.08)	0.175
Farmer	2.85 (1.08-12.38)	0.002	1.43 (0.28-7.16)	0.661
Merchant	1.38 (0.12-13.21)	0.873	1.31 (0.14-11.70)	0.804
Government employee	7.19(0.65-73.21)	0.054	7.16(0.71-72.72)	0.095
NGO employee	1.23 (0.19-5.29)	0.765	1.13 (0.21-5.16)	0.883
Casual laborer	5.69 (0.49-48.13)	0.541	5.17 (0.58-45.68)	0.139
Others			Reference	
**Time to reach the health facility**				
<1 hour	4.92 (1.01-11.61)	<0.001	2.57 (0.82-8.09)	0.105
1-2 hours	5.13 (1.23-14.37)	<0.001	2.89 (0.98-8.50)	0.053
More than 2 hours	Reference		Reference	
**Lost to follow up**				
Yes	5.23 (1.26-8.11)	0.003	3.24 (1.06-5.90)	0.036
No	Reference		Reference	
**Any comorbidity?**				
Yes	2.98 (1.12-5.09)	<0.001	2.48 (1.31-4.68)	0.005
No	Reference		Reference	
**Compliance with medications**				
Yes	3.58 (1.17-9.28)	0.045	1.50 (0.65-3.44)	0.332
No	Reference		Reference	

## Discussion

The objective of this research was to assess the prevalence of uncontrolled type 2 diabetes mellitus and identify its factors associated with type 2 diabetes patients in Kirehe District, Rwanda. The results of this study showed that 72.6% of patients in Kirehe District Health Facilities had uncontrolled type 2 diabetes mellitus. Being lost to follow-up at a non-communicable diseases clinic and having comorbidity were two factors significantly associated with uncontrolled type 2 diabetes mellitus among research participants. Patients who had ever been lost to follow-up were more likely to have uncontrolled type 2 diabetes mellitus compared to those who regularly visited the health facility for care and follow-up. Patients who had comorbidities to diabetes were more likely to have uncontrolled type 2 diabetes mellitus compared to those who did not have comorbidities. This prevalence was greater than the ones found in research conducted in the United States of America (1.7%) [12] and South Africa (11.4%) [13]; this means that rich countries have high-quality care given to their populations where some families have their own doctors and other afford the cost of access to quality care. The prevalence in this study is in line with other studies conducted in Bangladesh (61.3%) [14], Ethiopia (59.2%) [15] and Spain (57%) [16]. Even though the prevalence of this study is high, it stays less than the prevalence of uncontrolled type 2 diabetes mellitus in a study done in Ghana (86.4%) and Kenya (93%) [11]. According to health standards, these countries (Rwanda, Ethiopia, Bangladesh, and Spain) are not developed in terms of having enough healthcare providers and accessing all health services at a time without any constraints compared to developed countries such as the United States of America.

This study found that patients who had ever been lost to follow-up were more likely to have uncontrolled type 2 diabetes mellitus compared to those who regularly visited the health facility for care and follow-up. This result was in line with the one from a study done in Ghana [17]. This is explained by the fact that when a patient on diabetes medications didn´t show up at the health facility in a non-communicable diseases clinic more than three months from the previous visit, he/she didn´t take medications and laboratory checkups of blood sugar levels were not done, then diabetes has to stay uncontrolled. Patients who had comorbidities to diabetes were more likely to have uncontrolled type 2 diabetes mellitus compared to those who did not have comorbidities. In this study, having comorbidity was associated with uncontrolled type 2 diabetes mellitus and this was also found in studies conducted in Kenya [11] and Ghana [17]; when a patient with type 2 diabetes mellitus suffers from another disease, the situation worsens. The most common comorbidity is high blood pressure.

To mitigate patients´ loss to follow-up in non-communicable diseases (NCDS) clinics at health facilities and comorbidity with diabetes, the study results imply the recommendations at two levels of the health system in Kirehe District: elaborate an action plan to trace patients who are lost to follow-up in diabetes program in the whole District by working with the leaders and NCD nurses of all health centers, regularly screen all patients with diabetes for probable comorbidities such hypertension and regularly mentor and regularly supervise the 17 Health Centers to enhance NCD nurses with skills needed to reduce the rate of uncontrolled diabetes in Kirehe District are measures suggested to Kirehe District Hospital to deal with uncontrolled type 2 diabetes mellitus in the catchment area. The seventeen health centers were also recommended to train new nurses on diabetes diagnosis, management, follow-up and screening of diabetes probable comorbidities, to conduct regular home visits to trace and prevent loss to follow-up, and to retrain nurses on health education and counseling to diabetic patients about adherence, compliance, and diet restrictions. This study was limited in the way that it was carried out in one of the thirty districts that compose Rwanda as a country which limits the generalization of findings to the whole country even though it was conducted in rural area where more people are residing. The strength of this study is that it was conducted in all health facilities of the Kirehe District which makes to draw recommendations for the whole district.

## Conclusion

The prevalence of uncontrolled type 2 diabetes mellitus is still high; this requires more efforts to educate and counsel patients suffering from diabetes about appropriate diets, adherence to medications, respect of visiting non-communicable disease (NCDs) clinics for regular checkups, screening of probable comorbidities in order to prevent diabetes to become uncontrolled and its complications. Being defaulters and having comorbidities were significantly associated with uncontrolled T2DM among participants. Elaborate an action plan to trace patients who are lost to follow up in diabetes programs in the whole District by working with the leaders and NCD nurses of all health centers, regularly screen all patients with diabetes for probably comorbidities such hypertension and regularly mentor and regularly supervise the 17 Health Centers to enhance NCD nurses with skills needed to reduce the rate of uncontrolled diabetes in Kirehe District are measures suggested to Kirehe District Hospital to deal with uncontrolled type 2 diabetes mellitus in the catchment area. Health centers were also recommended to train new nurses on diabetes diagnosis, management, follow-up, and screening of diabetes probable comorbidities, to conduct regular home visits to trace and prevent loss to follow-up, and to retrain nurses on health education and counseling to diabetic patients about adherence, compliance, and diet restrictions.

### What is known about this topic

- *The prevalence of uncontrolled type 2 diabetes mellitus among patients in different countries other than Rwanda;*
- *Factors associated with uncontrolled type 2 diabetes mellitus among patients in different countries other than Rwanda.*

### What this study adds

- *The prevalence of uncontrolled type 2 diabetes mellitus among patients in Kirehe District Health Facilities, Eastern Province of Rwanda;*
- *Factors associated with uncontrolled type 2 diabetes mellitus among patients in Kirehe District Health Facilities, Eastern Province of Rwanda.*
